# Achieving Network Level Privacy in Wireless Sensor Networks^†^

**DOI:** 10.3390/s100301447

**Published:** 2010-02-26

**Authors:** Riaz Ahmed Shaikh, Hassan Jameel, Brian J. d’Auriol, Heejo Lee, Sungyoung Lee, Young-Jae Song

**Affiliations:** 1 Department of Computer Engeering, Kyung Hee University, Global Campus, Korea; E-Mails: riaz289@acm.org (R.A.S.); dauriol@oslab.khu.ac.kr (B.J.d’A.); yjsong@khu.ac.kr (Y.J.S.); 2 Computing Department, Macquarie University, NSW, Australia; E-Mail: hasghar@science.mq.edu.au; 3 Department of Computer Science & Engeering, Korea University, Seoul, Korea; E-Mail: heejo@korea.ac.kr

**Keywords:** anonymity, eavesdropping, hop-by-hop trace back, privacy, routing, wireless sensor networks

## Abstract

Full network level privacy has often been categorized into four sub-categories: *Identity*, *Route*, *Location* and *Data* privacy. Achieving full network level privacy is a critical and challenging problem due to the constraints imposed by the sensor nodes (e.g., energy, memory and computation power), sensor networks (e.g., mobility and topology) and QoS issues (e.g., packet reach-ability and timeliness). In this paper, we proposed two new identity, route and location privacy algorithms and data privacy mechanism that addresses this problem. The proposed solutions provide additional trustworthiness and reliability at modest cost of memory and energy. Also, we proved that our proposed solutions provide protection against various privacy disclosure attacks, such as eavesdropping and hop-by-hop trace back attacks.

## 1. Introduction

With the spreading application of Wireless Sensor Networks (WSNs) in various sensitive areas such as health-care, military, habitat monitoring, *etc*, the need to ensure security and privacy is becoming imperatively important. For example, in battlefield application scenario, “the location of a soldier should not be exposed if he initiates broadcast query” [1]. In the meantime, query must be transferred to the destination in an encrypted manner via only trusted en-route nodes. Similarly, in habitat monitoring application scenarios, such as Great Duck Island [2] or Save-the-panda application [3] where large numbers of sensor nodes are deployed to observe the vast habitat of ducks and pandas, an adversary can try to capture the panda or duck by back-tracing the routing path until it reaches the source sensor nodes. Therefore, in order to prevent the adversary from back-tracing, the route, location and data privacy mechanisms must be enforced. With respect to these application scenarios, network level privacy has often been categorized into four categories:

1. Sender node identity privacy: no intermediate node can get any information about who is sending the packets except the source, its immediate neighbors and the destination,
2. Sender node location privacy: no intermediate node can have any information about the location (in terms of physical distance or number of hops) about the sender node except the source, its immediate neighbors and the destination,
3. Route privacy: no node can predict the information about the complete path (from source to destination). Also, a mobile adversary gets no clue to trace back the source node either from the contents and/or directional information of the captured packet(s), and
4. Data packet privacy: no node can see the information inside in a payload of the data packet except the source and the destination.

Existing privacy schemes such as [1, 3–7], that have specifically been proposed for WSNs only provide partial network level privacy. Providing a full network level privacy is a critical and challenging issue due to the constraints imposed by the sensor nodes (e.g., energy, memory and computation power), sensor network (e.g., mobility and topology) and QoS issues (e.g., packet reach-ability and trustworthiness). Thus, an energy-efficient privacy solution is needed to address these issues.

In order to achieve this goal, we incorporate basic design features from related research fields such as geographic routing and cryptographic systems. To our knowledge, we propose the first full network level privacy solution for WSNs. Our contribution lies in following features.

- A new Identity, Route and Location (IRL) privacy algorithm is proposed that ensures the anonymity of source node’s identity and location. It also assures that the packets will reach their destination by passing through only trusted intermediate nodes.
- A new reliable Identity, Route and Location (r-IRL) privacy algorithm is proposed, which is the extension of our proposed IRL algorithm. This algorithm has the ability to forward packets from multiple secure paths to increase the packet reach-ability.
- A new data privacy mechanism is proposed, which is unique in the sense that it provides data secrecy and packet authentication *in the presence of identity anonymity*.

Our solutions collectively provide protection against various privacy disclosure attacks such as eavesdropping and hop-by-hop trace-back attacks. Also, our solutions are lightweight, hence consume modest memory and energy.

The rest of this paper is organized as follows: Section 2. contains related work, Section 3. articulates the network model, assumptions and adversary model. Section 4. describes the proposed privacy schemes, Section 5. consists of analysis and evaluation, and Section 6. concludes the paper.

## 2. Related Work

### 2.1. Privacy Schemes

A number of a privacy schemes such as [1, 3–7] have been proposed for WSNs that are discussed below.

C. Ozturk *et al*. [3] proposed a phantom routing scheme for WSNs, which helps to prevent the location of a source from the attacker. In this scheme, each message reaches the destination in two phases: 1) a walking phase, in which the message is unicasted in a random fashion within first *h_walk_* hops, 2) after that, the message is flooded using the baseline flooding technique. The major advantage of their scheme is the source location privacy protection, which improves as the network size and ntensity increase because of high path diversity. But on the other hand, if the network size increases, the flooding phase will consume more energy. This scheme does not provide identity privacy. Also, it is unable to provide data secrecy in the presence of identity privacy.

P. Kamat *et al*. [4] proposed a phantom single-path routing scheme that works in a similar fashion as the original phantom routing scheme [3]. The major difference between these two schemes is that after the walking phase, a packet will be forwarded to the destination via a single path routing strategy such as the shortest path routing mechanism. This scheme consumes less energy and requires slightly higher memory as compared to first one. This scheme also does not provide identity privacy. Also, it is unable to provide data secrecy in the presence of identity privacy.

S. Misra and G. Xue [5] proposed two schemes: Simple Anonymity Scheme (SAS) and Cryptographic Anonymity Scheme (CAS) for establishing anonymity in clustered WSNs. The SAS scheme use dynamic pseudonyms instead of true identity during communications. Each sensor node needs to store a given range of pseudonyms that are non-contiguous. Therefore, the SAS scheme is not memory efficient. On the other hand, the CAS scheme uses keyed hash functions to generate pseudonyms. This scheme is memory efficient as compare to the SAS but it requires more computation power. The authors do not propose any routing scheme. Sender node may always send packets to the destination via shortest path. In that case, for an adversary who is capable of performing hop-by-hop trace back (with the help of direction information) can find out the location of the source node.

Y. Xi *et al*. [1] proposed a Greedy RandomWalk (GROW) scheme to protect the location of the source node. This scheme works in two phases. In a first phase, the sink node will set up a path through random walk with a node as a receptor. Then the source node will forward the packets towards the receptor in a random walk manner. Once the packet reaches at the receptor, it will forward the packet to the sink node through the pre-established path. Here receptor is acting a central point between the sink and the source node for every communication session. A criterion of selecting a trustworthy receptor is essential, however not defined in the author’s work.

Y. Ouyang *et al*. [7] proposed a Cyclic Entrapment Method (CEM) to minimize the chance of an adversary in finding out the location of the source node. In the CEM, when the message is sent by the source node to the base station, it will activate the predefined loop(s) along the path. An activation node will generate the fake message and forwarded it towards the loop, and original message is forwarded to the base station via specific routing protocol such as shortest path. Energy consumption in the CEM scheme is mainly dependent on the number of existing loops in the path and their size.

### 2.2. Geographic Routing Schemes

Our proposed privacy solutions incorporate the basic design features from geographic routing schemes [6, 8–10] that are discussed below.

M. Zorzi and R. R. Rao [8, 9] proposed Geographic Random Forwarding (GeRaF) scheme for ad hoc and sensor networks. This scheme is based on broadcast transmission and the sender only requires the position of its own and the destination. All active neighborhood nodes who receive the packet will go through the contention phase. Once the contention phase is complete, the winner (the node that is closest to the destination) will relay the packet using the same mechanism. This process will repeat until the destination becomes one-hop away. The authors assumed that all nodes in the neighborhood do not remain active all the time. Due to the dynamics of the sleep modes, different sets of potential relays will be available. However, mostly the potential route is close to the same or shortest route, which makes easier for an adversary to trace back to the sender.

A. Capone *et al*. [10] proposed Simple Forwarding over Trajectory (SiFT) scheme. This scheme is based on broadcast transmission and does not maintain neighborhood positions and states. Each node who receives the packet will make the decision of forwarding that packet based only on its own position, the position of a transmitter and the trajectory. The difference between the GeRaF [8, 9] and the SiFT scheme is that, the GeRaF does not use trajectories but the position of the destination. If nodes are static then similar to the GeRaF the potential route is close to the same or shortest route, which makes it easier for an adversary to trace back to the sender.

A. D. Wood *et al*. [6] have proposed a configurable secure routing protocol family called Secure Implicit Geographic Forwarding (SIGF) for WSNs. The SIGF is based on the Implicit Geographic Forwarding (IGF) protocol [11], in which a packet is forwarded to the node that lies within the region of 60° sextant, centered on the direct line from the sender to the destination. The SIGF protocol provides some aspects of networks privacy such as data, route and location privacy, but it does not provide identity privacy. Another limitation of the SIGF protocol is that, when there is no trusted node within a forwarding area (assuming 60° sextant), it will forward the packet to an un-trusted node. So, the reliability of the path is affected.

Table 1 compares the proposed privacy preserving schemes. It clearly shows that none of the schemes currently provide full network level privacy.

## 3. Network, Assumptions and Adversary Model

### 3.1. Network Model

**Figure 1.**
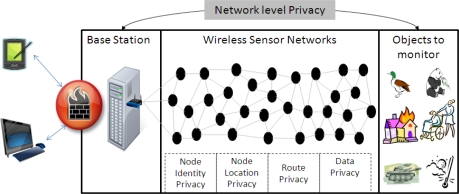
Typical WSN scenario.

A wireless sensor network (WSN) is composed of large number of small sensor nodes that are of limited resource and densely deployed in an environment. Whenever end users require information about any event related to some object(s), they send a query to the sensor network via the base station. And the base station propagates that query to the entire network or to a specific region of the network. In response to that query, sensor nodes send back required information to the base station. A typical wireless sensor network scenario is shown in Figure 1. Links are bidirectional. Also, sensor nodes use IEEE 802.11 standard link layer protocol, which keeps packets in its cache until the sender receives an acknowledgment (ACK). Whenever a receiver (next hop) node successfully receives the packet it will send back an ACK packet to the sender. If the sender node does not receive an ACK packet during predefined threshold time, then the sender node will retransmit that packet.

### 3.2. Assumptions

For reason of scalability, it is assumed that no sensor node needs to know the global network topology, except that it must know the geographical location of its own, its neighboring nodes and the base station. In order to find out the location information, any proposed mechanism could be used, such as [12, 13].

It is assumed that each sensor node in the network can share a unique secret key with the base station [14, 15]. These keys are periodically updated. The public key of the base station is also assumed known to all the nodes in the network. Sensor nodes do not require their own public and private keys; because computation cost of public and private keys is generally consider being high. However, many researchers [16, 17] have shown the feasibility of using public key cryptography in wireless sensor networks. It is also assumed that sensor nodes are capable of performing encryption and decryption of the data by using any cipher algorithm such as DES, AES *etc*. This provides an additional layer of security.

This paper only focuses on the development of a prevention strategy against network level privacy disclosure attacks, such as eavesdropping, traffic analysis and hop-by-hop trace back attacks. Other general attacks, such as flooding attacks, could be detected and prevented by using any IDS scheme proposed for WSNS.

### 3.3. Adversary Model

We have assumed that an adversary can mostly perform passive attacks (like eavesdropping [18], and traffic analysis), since such attacks helps to conceal the adversary’s presence in the network. Nevertheless, the adversary is also capable of performing some active attacks like fabrication and packet drop attacks. We also assumed that the adversary is both device-rich and resource-rich [4]. These characteristics are defined below.

- Device-rich: the adversary is equipped with devices like antenna and spectrum analyzers, so that the adversary can measure the angle of arrival of the packet and received signal strength. These devices will help the adversary to find out the immediate sender of the packet and move to that node. This kind of hop-by-hop trace back mechanism will be carried out by the adversary until the actual sender node is reached.
- Resource-rich: the adversary has no resource constraint in computation power, memory or energy.

It is also assumed that the adversary has some basic domain knowledge like the range of identities assigned to the sensor nodes, the public key of the base station and information about the cipher algorithms used in the network. However, adversary has no knowledge which identity is physically associated with which node.

A detection and prevention strategy against non-privacy disclosure attacks at various layers such as jamming attacks is out of the scope of this paper. However, trust management methodology (Section 4.1) that we adopted in this paper is useful to detect and prevent some non-privacy disclosure threats such as, black hole attack, sink hole attack, and selective forwarding or gray hole attack.

## 4. Proposed Scheme

### 4.1. Concepts and Definitions

In our proposed algorithms, we have used two notions: direction and trust. Both these notions (direction and trust) are used to provide reliable (non-malicious and non-faulty) secure paths for achieving robust route privacy. Direction information will help to forward packet to the destination in a timely manner and trust will help to forward the packets via reliable nodes. Detail definitions of both notions are given below.

**Figure 2.**
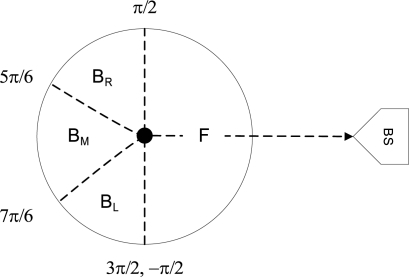
Neighbor node classification

**Direction:** The first notion used in our algorithms is that of direction. The physical location of the base station is the reference point for each sensor node. Based on this reference point, each node classifies its neighboring nodes into four categories: (1) forward neighboring nodes (*F*), (2) right side backward neighboring nodes (*B_r_*), (3) left side backward neighboring nodes (*B_l_*), and (4) middle backward neighboring nodes (*B_m_*). The objective of this categorization is to provide more path diversity as discussed in Section 4.2. A node *x* classifies its neighboring node *y* in following fashion:

(1) $$C_{x,y}=\{\begin{array}{cc} F & \frac{-\pi }{2}\leq \theta \leq \frac{\pi }{2}\\ B_{r} & \frac{\pi }{2}<\theta \leq \frac{5\pi }{6}\\ B_{m} & \frac{5\pi }{6}<\theta \leq \frac{7\pi }{6}\\ B_{l} & \frac{7\pi }{6}<\theta <\frac{3\pi }{2} \end{array}$$

where *θ* is the angle between the node *x* and its neighboring node *y* with respect to the line joining node *x* and the base station as shown in Figure 2.

**Trust:** The second notion used in our algorithms is that of trust. The definition of a trust here is based on our other paper [19] and restated here.

A node can be classified into one of the three categories [20]: trustworthy, untrustworthy, and uncertain. A node is considered trustworthy if it interacts successfully most of the time with the other nodes. A node is considered untrustworthy if it tries to do as many unsuccessful interactions as possible with the other nodes. An untrustworthy node could be a faulty [21] or malicious node. A node is considered uncertain if it performs both successful and unsuccessful interactions. Detailed definition of the successful and unsuccessful interactions and trust calculation methodology is available in our paper [22] and provided here in a simplified form.

A sender will consider an interaction successful if the sender receives confirmation that the packet is successfully received by the neighbor node and forwarded towards the destination in an unaltered fashion. The first requirement of successful reception is achieved on the reception of the link layer acknowledgment (ACK). The second requirement of forwarding towards the destination is achieved with the help of enhanced passive acknowledgment (PACK) by overhearing the transmission of a next hop on the route, since they are within the radio range [23]. If the sender node does not overhear the retransmission of the packet within a threshold time from its neighboring node or if the overheard packet is found to be illegally fabricated (by comparing the payload that is attached to the packet), then the sender node will consider that interaction as unsuccessful.

With the help of this simple approach, several attacks can be prevented, *i.e.*, the black hole attack is straightforwardly detected when malicious node drops the incoming packets and keeps sending self generated packets [24]. Similarly, sink hole attack [25], an advanced version of the black hole attack, is also easily detectable by looking at the passive acknowledgment. Likewise, the selective forwarding attack [26] and gray-hole attack [27] can also be eliminated with the aid of above mentioned approach.

Based on these successful and unsuccessful interactions node *x* can calculate the trust value of node *y* in following fashion:

(2) $$T_{x,y}=\left[100\left(\frac{S_{x,y}}{S_{x,y}+U_{x,y}}\right)\left(1-\frac{1}{S_{x,y}+1}\right)\right]$$

where [.] is the nearest integer function, *S_x,y_* is the total number of successful interactions of node *x* with *y* during time *δt*, and *U_x,y_* is the total number of unsuccessful interactions of node *x* with *y* during time *δt*. After calculating trust value, a node will quantize trust into three states as follows:

(3) $$Mp(T_{x,y})=\left\{\begin{array}{cc} \text{trustworthy} & 100-f\leq T_{x,y}\leq 100\\ \text{uncertain} & 50-g\leq T_{x,y}<100-f\\ \text{untrustworthy} & 0\leq T_{x,y}<50-g \end{array}\right\}.$$

where, *f* represents half of the average values of all trustworthy nodes and *g* represents one-third of the average values of all untrustworthy nodes. Both *f* and *g* are calculated as follows:

(4) $$f_{j+1}=\{\begin{array}{cc} \left[\frac{1}{2}\left(\frac{\sum_{i\in R_{x}}T_{x,i}}{\left|R_{x}\right|}\right)\right] & 0<\left|R_{x}\right|\leq n-1\\ f_{j} & \left|R_{x}\right|=0 \end{array}$$

(5) $$g_{j+1}=\{\begin{array}{cc} \left[\frac{1}{3}\left(\frac{\sum_{i\in M_{x}}T_{x,i}}{\left|M_{x}\right|}\right)\right] & 0<\left|M_{x}\right|\leq n-1\\ g_{j} & \left|M_{x}\right|=0 \end{array}$$

where [.] is the nearest integer function, *R_x_* represents the set of trustworthy nodes for node *x*, *M_x_* the set of untrustworthy nodes for node *x*, and *n* is the total number of nodes that contains trustworthy, untrustworthy and uncertain nodes. The initial trust values of all nodes are 50, which represents the uncertain state. Initially *f* and *g* are equal to 25 and 17 respectively, although other values could also be used by keeping the following constraint intact: *f_i_* − *g_i_* ≥ 1, which is necessary for keeping the uncertain zone between a trusted and untrustworthy zone. The values of *f* and *g* are adaptive. During the steady-state operation, these values can change with every passing unit of time which creates dynamic trust boundaries. At any stage, when |*R_x_*| or |*M_x_*| becomes zero, the value of *f_j_*_+1_ or *g_j_*_+1_ remains the same as the previous values (*f_j_* and *g_j_*). The nodes whose values are above 100 − *f* will be declared as trustworthy nodes (Equation 3), and nodes whose values are lower than 50 − *g* will be consider as untrustworthy nodes (Equation 3). After each passage of time, Δ*t*, nodes will recalculate the values of *f* and *g*. This trust calculation procedure will continue in this fashion.

The time window length (Δ*t*) could be made shorter or longer based on the network analysis scenarios. If Δ*t* is too short, then the calculated trust value may not reflect the reliable behavior. On the other hand, if it is too long, then it will consume too much memory to store the interaction record at the sensor node. Therefore, various parameters can be used to adjust the length of Δ*t*.

### 4.2. Identity, Route, and Location Privacy (IRL)

Our proposed identity, route and location privacy scheme works in two phases. The first is neighbor node state initialization phase, and the second is routing phase.

*Route Privacy:* In initialization phase, let the node *i* have *m* neighboring nodes in which *t* nodes are trusted. So, 0 ≤ *t* ≤ *m* and *M*(*t*) = *M*(*t_F_*) ∪ *M*(*t*_*B*_*r*__) ∪ *M*(*t*_*B*_*l*__) ∪ *M*(*t*_*B*_*m*__). Here *M*(*t_F_*), *M*(*t*_*B*_*r*__), *M*(*t*_*B*_*l*__), and *M*(*t*_*B*_*m*__) represent the set of trusted nodes that are in the forward, right backward, left backward, and middle backward directions, respectively. These neighbor sets (*M*(*t_F_*), *M*(*t*_*B*_*r*__), *M*(*t*_*B*_*l*__), and *M*(*t*_*B*_*m*__)) are initialized and updated whenever a change occur in neighborhood. For example, the entrance of a new node, change of a trust value, *etc*.

Whenever a node needs to forward a packet, the routing phase (Algorithm 1 for source node and Algorithm 2 for intermediate node) of IRL algorithm is called.

Whenever a source node (Algorithm 1) wants to forwards the packet, it will first check the availability of the trusted neighboring nodes in its forward direction set *M*(*t_F_*) (Line 2). If trusted nodes exists then it will randomly select one node as a next hop (Line 3) from the set *M*(*t_F_*) and forward the packet towards it (Lines 13:21). If there is no trusted node in its forward direction, then the source node will check the availability of a trusted node in the right (*M*(*t*_*B*_*r*__)) and left (*M*(*t*_*B*_*l*__)) backward sets. If the trusted nodes are available then the source node will randomly select one node as a next hop (Line 3) from these sets and forward the packet towards it (Lines 13:21). If the trusted node does not exist in these sets either, then the source node will randomly select (Line 8) one trusted node from the backward middle set (*M*(*t*_*B*_*m*__)) and forward the packet towards it (Lines 13:21). If there are no trusted nodes available in all of the sets then the packet will be dropped (Line 9:10).

**Algorithm 1:** IRL - Routing at Source Node.

1:	*prev_hop_* ← ∅; *next_hop_* ←∅;
2:	**if***M*(*t_F_*) ≠ ∅ **then**
3:	*next_hop_*(*k*) = Rand(*M* (*t_F_*));
4:	**else**
5:	**if***M*(*t*_*B*_*r*__) ∪ *M*(*t*_*B*_*l*__) ≠ ∅ **then**
6:	*next_hop_*(*k*) = Rand(*M* (*t*_*B*_*r*__) ∪ *M* (*t*_*B*_*l*__));
7:	**else if***M*(*t*_*B*_*m*__ ≠ ∅ **then**
8:	*next_hop_*(*k*) = Rand(*M* (*t*_*B*_*m*__));
9:	**else**
10:	Drop packet and Exit;
11:	**end if**
12:	**end if**
13:	Set *prev_hop_* = *my_id_*;
14:	Form pkt *p* = {*prev_hop_*; *next_hop_*; *seqID; payload*};
15:	Create Signature and save in buffer;
16:	Forward packet to *next_hop_*;
17:	Set timer $\Delta t=\frac{D}{d_{{\mathrm{next}}_{\mathrm{hop}}}}\times p_{t};$
18:	**while** Δ*t* = *true***do**
19:	Signature remains in buffer;
20:	**end while**
21:	Signature removed from buffer;

When an intermediate node (Algorithm 2) receives the packet (either from the source node or from another en-route node), it will first check whether the packet is new or old (Line 3). If it is new, then the node will first check the availability of the trusted node from the forward direction set (*M_F_*) excluding the *prev_hop_* node if it belongs to forward set (Line 13). If trusted nodes exists in the forward set then the node will randomly select any one trusted node as a next hop (Line 14) and forward the packet towards it (Line 45). If there is no trusted node available in the forward direction, then it will check to which set the sender of the packet belongs to. For example, If the packet, forwarded by a node, belongs to the right backward set (Line 16), then it will first check whether the left or middle backward sets contain any trusted nodes (Lines 17:18). If so, it will randomly select one node from those sets (Line 19) and forward the packet towards it (Line 45). If there is no trusted node in those two sets, then the node will randomly select a trusted node from the right backward set (*M*(*t*_*B*_*r*__)) excluding the one from which the node received the current packet (Lines 20:21) and forward the packet towards it (Line 45). Similar operations will be performed, if the packet, forwarded by a node, belongs to the left (Lines 25:33) and middle backward or forward (Lines 34:43) sets. An example IRL routing scenario is shown in Figure 3.

**Algorithm 2:** IRL - Routing at Intermediate Node.

1:	*next_hop_* ← ∅;
2:	*M_temp_* = ∅
3:	**if** Signature of new packet already exists in buffer **then**
4:	*M_temp_* = {*M_temp_*} + *LasttimePrev_hop_*
5:	*M_temp_* = {*M_temp_*} + *LasttimeNext_hop_*
6:	Set *counter* = *timesReceviedBefore* + 1;
7:	Remove signature from buffer;
8:	**if***counter* = 3 **then**
9:	Drop packet and exit;
10:	**end if**
11:	**end if**
12:	*M_temp_* = {*M_temp_*} + *prev_hop_*
13:	**if** (*M*(*t_F_*) − {*M*(*t_F_*) ∩ *M_temp_*}) ≠ ∅ **then**
14:	*next_hop_*(*k*) = Rand(*M*(*t_F_*) − {*M*(*t_F_*) ∩ *M_temp_*});
15:	**else**
16:	**if** packet came from *B_r_***then**
17:	*M_temp_*_1_ = *M*(*t*_*B*_*l*__) ∪ *M*(*t*_*B*_*m*__)
18:	**if***M_temp_*_1_ ≠ ∅ **then**
19:	*next_hop_*(*k*) = Rand(*M_temp_*_1_);
20:	**else if***M*(*t*_*B*_*r*__) ≠ ∅ **then**
21:	*next_hop_*(*k*) = Rand(*M*(*t*_*B*_*r*__) − {*M*(*t*_*B*_*r*__) ∩ *M_temp_*});
22:	**else**
23:	Drop packet and Exit;
24:	**end if**
25:	**else if** packet came from *B_l_***then**
26:	*M_temp_*_2_ = *M*(*t*_*B*_*r*__) ∪ *M*(*t*_*B*_*m*__)
27:	**if***M_temp_*_2_ ≠ ∅ **then**
28:	*next_hop_*(*k*) = Rand(*M_temp_*_2_ − {*M_temp_*_2_ ∩ *M_temp_*});
29:	**else if***M*(*t*_*B*_*l*__) ≠ ∅ **then**
30:	*next_hop_*(*k*) = Rand(*M*(*t*_*B*_*l*__) − {*M*(*t*_*B*_*l*__) ∩ *M_temp_*});
31:	**else**
32:	Drop packet and Exit;
33:	**end if**
34:	**else**
35:	*M_temp_*_3_ = *M*(*t*_*B*_*r*__) ∪*M*(*t*_*B*_*l*__)
36:	**if***M_temp_*_3_ ≠ ∅ **then**
37:	*next_hop_*(*k*) = Rand(*M_temp_*_3_ − {*M_temp_*_3_ ∩*M_temp_*});
38:	**else if***M*(*t*_*B*_*m*__) ≠ ∅ **then**
39:	*next_hop_*(*k*) = Rand(*M*(*t*_*B*_*m*__) − {*M*(*t*_*B*_*m*__)∩*M_temp_*});
40:	**else**
41:	Drop packet and Exit;
42:	**end if**
43:	**end if**
44:	**end if**
45:	Rest is same as Algorithm 1 from lines 13:21;

This routing strategy may result in the creation of a cycle (loop). However, due to the randomness in the selection of the next-hop and the presence of the different four direction sets, the probability of creation of any cycle is very low. Nevertheless, in order to fully avoid the occurrence of the cycles, each node (prior to forwarding of a packet) will save the signature of the packet in the buffer for the *δt* time, that is:

(6) $$\delta t=2\left(\frac{D}{d}\times p_{t}\right)$$

where *D* is the distance between the forwarding node and the base station, *d* is the distance between the forwarding node and the next hop, and *p_t_* is the propagation transfer time between the forwarding node and the next hop. This signature consists of two fields: (1) sequence number of the packet, and (2) the payload. The potential of the signature to compare and identify the same packet is detailed in the later section. Corresponding to this signature, three more fields are also stored in the buffer: (1) previous hop identity, (2) next hop identity where the packet is forwarded, and (3) counter, that tells how many times the same packet is received by the node. This information will later be used to get rid of any cycle. The size of the buffer is mainly dependent on the network traffic conditions. However, it is expected to be low due because the sensor nodes sent data either in periodic intervals or upon the occurrence of some event.

**Figure 4.**
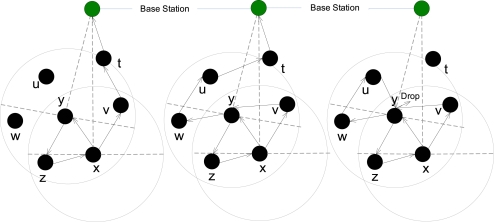
Three sample cycle detection and prevention scenarios.

If the node received the packet whose signature exists in the buffer (Algorithm 2, Lines 3:11), then including the previous hop node (Line 12), two other nodes will also be excluded from the selection of the next hop process: 1) the node from which last time the packet was received (Line 4) and 2) the node from which last time the packet was forwarded (line 5). If the same packet is received three times by the same node (Line 8) then the packet will be dropped (Line 9). Three sample scenarios of the loop creation, detection and prevention are shown in Figure 4. Creation of loops and traversing of the packets in the backward direction is not a completely negative effect. Rather, it provides positive effects in terms of strengthening the route and source location privacy, because these effects will helps to increase the safety period [3], which is the time for an adversary to reach at the source node.

*Identity Privacy*: Whenever a node receives the packet *p* from the source node or en-route node then the receiving node will replace the previous hop’s identity *prev_hop_* contained in the packet with its own (Algorithm 1, Line 13). After that, the node will get the next forwarding node *next_hop_* (as described earlier) and update the header of the packet *p* = {*prev_hop_, next_hop_, payload*} (Line 14). After modification of the two header fields, the node will forward the packet (Line 16). In this way, all the intermediate forwarding nodes replace the source and next hop’s identity contained in the packet *p*. This process will go on until the packet reaches the base station.

*Location Privacy*: The neighboring nodes which are in each other’s radio range can easily approximate the location of each other by measuring the received signal strength and the angle of arrival [28]. If the adversary is within the range of the source node, then adversary can easily estimate the location of the source. Once the packet has crossed the radio range of the original source node, then becomes very difficult for an attacker to estimate the location of the node either in terms of the physical distance or in terms of the number of hops of an original source node. The main reason for this is that the path selection is random and packets are forwarded by only trusted nodes which only contain the information of the last and the next hop.

### 4.3. Reliable Identity, Route, and Location Privacy (r-IRL)

It is also possible that some applications require more reliability in terms of packet reach-ability; and the packet could be dropped due to either network congestion or malicious behavior of an en-route node. Thus, in order to achieve more reliability, the packet should be forwarded from multiple paths simultaneously, which will give trustworthiness in the sense that at least the packet should reach the base station by any one of the paths, although, this may increase some communication overhead.

Our reliable IRL (r-IRL) algorithm is the extended version of our proposed IRL algorithm, in which we introduce one more parameter, reliability *r*. The source node *i* will multi-cast a packet to all *r* randomly selected neighboring trusted nodes that are in the forward direction. If there are no adequate trusted nodes present in the forward direction, then it will select the remaining trusted nodes from the backward direction. The rest of the mechanism of the r-IRL algorithm is the same as the IRL algorithm.

### 4.4. Data Privacy

The payload contains the identity of the source node (*ID_x_*) and the actual data (*d*). Identity is encrypted with the public key ( $k_{\mathrm{bs}}^{+}$) of the base station and data is encrypted with the secret key (*k_x,bs_*) shared between the sender node and the BS. Both are appended with the payload as shown below:

$$\mathrm{payload}=[E(ID_{x},k_{\mathrm{bs}}^{+}),\;E(d,k_{x,bs})]$$

If we assume that the adversary knows the range of identities assigned to the sensor nodes, public key of the base station and information about cipher algorithm used in the network, an adversary can then successfully obtain the identity of the source by performing simple brute-force search attack [29] by comparing the pattern of encrypted identity with a known range of identities. Therefore in order to provide protection against brute-force search attack, we append a random number (*R_n_*) (equivalent to the size of identity) with the identity of a node and then perform encryption. Now the payload is:

$$\mathrm{payload}=[E(ID_{x}\|R_{n},k_{\mathrm{bs}}^{+}),\;E(d,k_{x,bs})]$$

where ‖ is the append operation. Inclusion of random number may introduce additional computational overhead. However, the amount of overhead is mainly dependent on random number generation technique. Recently, very nice random generation techniques have been specially designed for low power sensor networks, such as [30, 31]. These techniques could be used to generate random number for each packet. Also, overall computational overhead is dependent on the number of packets generated by the sensor nodes. Mostly, sensor nodes are event driven or query driven [32]. Therefore, amount of traffic is usually kept low as compared to traditional networks.

Our proposed data privacy approach provides several benefits. Firstly, data secrecy is achieved in the presence of identity anonymity. This feature is not available in earlier proposed privacy schemes. Secondly, the base station will receive both the identity of the actual source node and message authentication. If the packet has been successfully decrypted with the shared secret key, it means that packet is received from genuine sensor node.

## 5. Analysis and Evaluation

### 5.1. Security Resiliency Analysis

Suppose we have an adversary 𝒜 who strives to defeat our privacy protocols and guess the original source node. We will distinguish between two kinds of nodes. A source node is the original sender of a packet *q* and a forwarding node is the node that forwards a packet to another node until it reaches the destination. Hence the source node is also a forwarding node. The adversary’s goal is to find out the source node. This analysis assumes that we are using IRL algorithm including our proposed data privacy mechanism. So if the adversary sees a packet, it will trivially know the identity of the last forwarding node (which could possibly be the sender node).

We will deal with separate cases. Case 1 is when the adversary is close to the base station and can eavesdrop on any packet received by the base station. Case 2 deals with the case when the adversary can see any packet within the radio range of a particular node. Case 3 extends this into two or more nodes.

An adversary will try to solve the following problem: Given a packet *q* and a subset of nodes *N′*, find out the sender node *s*. In other words, the algorithm for the adversary takes two inputs and outputs a node *s′*; Namely 𝒜(*q,N′*) = *s′*. If *s′* = *s*, the adversary succeeds in defeating our protocol. We have to find: Pr[𝒜(*q,N′*) = *s*], which is the probability for an adversary to find out the sender node. Our assumption is that, from an adversarial perspective, all nodes are equally likely to be senders of a packet. This does not necessarily mean that the network traffic is uniformly distributed. Notice that if the adversary knows beforehand which nodes are more likely to send packets, then no privacy preserving method can prevent the adversary from guessing the most likely senders, since this constitutes the adversary’s a priori knowledge.

**Notations and definitions:** Denote a generic node by *m*. The set of neighbors of *m* is denoted by *N_m_*, which also includes *m* itself. The number of forward and backward nodes of *m* is denoted by *m_f_* and *m_b_* respectively. If a node *a* is a backward node of *m*, then we denote it as *a* → *m*. We say that a node *a* is in the backward set of node *m*, if *a* → *a*_1_ → . . . *a_r_* → *m*, for some nodes *a*_1_, . . . *a_r_* where *r ≥* 0. For compact notation we will denote this as *a* →*^r^* *m*, if the IDs of the intermediate nodes are not significant. We will also use the notation →*^r^* *m* to denote a generic node, who is *r* links (hops) away from *m*. Define the backward set *C_m_* of *m* as *C_m_* = {*a|a* →*^r^* *m, r ≥* 0}, that is the set of all the possible nodes such that they have a forward link to *m*. Denote the base station as *B*. It will also be seen as another node. Let the total number of nodes in the network excluding the base station be *N*. We will use the term “adversary is in possession of a node” to indicate that the adversary can passively listen to any communication within the radio range of that node.

**Claim 1:** Suppose 𝒜 is in possession of *B*. Let *B_b_* be the number of backward nodes of the base station (nodes one hop away from the base station). Then for any packet *q* received by *B* and for large enough *N*:

(7) $$\text{Pr}[𝒜(q,N)=s]\approx \frac{B_{b}+1}{N}$$

*Proof* : The adversary can always know the ID of the last forwarding node. Let *B_b_* be the number of backward nodes to the base station. The packet could only have come from one of the nodes in *N_B_* − {*B*} (which only contains backward nodes to *B*). Since the nodes are just a hop away from the BS, so they will not send the packet to another node. Hence for large *N* we have:

$$\begin{array}{c} \text{Pr}\;[𝒜(q,N)=s]=\text{Pr}\;[𝒜(q,N)=s|s\in N_{B}-\{B\}]\times \text{Pr}\;[s\in N_{B}-\{B\}]+\\ \text{Pr}\;[𝒜(q,N)=s|s\notin N_{B}-\{B\}]\;\text{Pr}\;[s\notin N_{B}-\{B\}]\\ =1\cdot \frac{B_{b}}{N}+\frac{1}{N-B_{b}-1}\left(1-\frac{B_{b}}{N}\right)\\ \approx \frac{B_{b}}{N}+\frac{1}{N-B_{b}}\left(1-\frac{B_{b}}{N}\right)=\left(\frac{B_{b}+1}{N}\right) \end{array}$$

Now let us assume that 𝒜 is in possession of a node *m* in the network. The following probability estimate gives an upper bound of the probability of success of the adversary. It is an upper bound since it does not include the possibility of a packet sent backwards. When a packet is sent backwards over one or many hops, the probability of success of the adversary decreases since there would be more possible nodes. Thus in this scenario our result would be like an upper bound on the adversary’s limitations.

**Claim 2:** Suppose 𝒜 is in possession of a node *m*. Let *c* = |*C*_→^2^*m*_| denote the number of backward nodes in backward set *C*_→^2^*m*_ of some node →^2^*m*. Then,

(8) $$\text{Pr}\;[𝒜(q,N)=s]\leq \frac{m_{f}+m_{b}+1}{N}+\frac{1}{c+1}\left(1-\frac{m_{f}+m_{b}+1}{N}\right)$$

*Proof* : Since the adversary is in possession of a node *m*, it knows its backward and forward nodes. Furthermore, if any of these nodes including the node *m* itself is the sender of a packet *q*, then the adversary will know. This is true since the adversary can see all incoming packets to the node *m* and to its neighbor nodes (the forward and the backward nodes). Thus it can see if the payload of *q* is not equal to the payload of any *q′* being received by these nodes in a given interval of time. If this is the case, then the adversary will know the sender.

Now if none of the nodes in *N_m_* are the senders, then the packet was forwarded by a node *i* that is two hops away from *m*. The adversary knows the ID of that node through the packet *q*. Thus the adversary makes a list of all the possible backward nodes in the backward set of *i*. Let that number be denoted by *c*. Notice that node *i* could also be the possible sender. Hence the total number of possible senders would be *c* + 1. We have:

$$\begin{array}{c} \text{Pr}\;[𝒜(q,N)=s]=\;\text{Pr}\;[𝒜(q,N)=s|s\in N_{m}]\;\text{Pr}\;[s\in N_{m}]+\\ \text{Pr}\;[𝒜(q,N)=s|s\notin N_{m}]\;\text{Pr}\;[s\notin N_{m}]\\ \leq \frac{m_{f}+m_{b}+1}{N}+\frac{1}{c+1}\left(1-\frac{m_{f}+m_{b}+1}{N}\right) \end{array}$$

Now, suppose the adversary is in possession of two nodes at the same time; *m*_1_ and *m*_2_. We can safely assume that *N*_*m*_1__ ∩ *N*_*m*_2__ = *φ*, since it would be more advantageous to the adversary to cover nodes with non-overlapping radio ranges. The adversary will always know whenever any node in *N*_*m*_1__ or *N*_*m*_2__ is the sender of a packet. How about the case when they are not the senders? There could be two possible cases: without loss of generality, first assume that *m*_2_ ∈ *C*_*m*_1__. If the packet *q* was received by some node in *N*_*m*_1__ and was received by some node in *N*_*m*_2__ before, then the adversary had already checked it when the packet was sent to a node in *N*_*m*_1__. Thus the adversary need only check packets received in *N*_*m*_1__ that were not received by *N*_*m*_2__. In this case, the sender cannot be in *N*_*m*_2__. In any case, the adversary has to find out the backward sets of →^2^ *m*_1_ or →^2^ *m*_2_, depending on where the packet was received. Since, in the adversary’s knowledge, all nodes are equally likely to be senders, the probability of a packet being received at the two sets is the same. In case *m*_2_ ∉ *C*_*m*_1__, then the adversary has no real advantage except that it can see packets at two disjoint locations in the network. Thus we only state the case when *m*_2_ ∈ *C*_*m*_1__. We have the following result:

**Claim 3:** Suppose the adversary is in possession of two nodes *m*_1_ and *m*_2_. Assume further that *m*_2_ ∈ *C*_*m*_1__. Let *c*_1_ = |*C*_→^2^*m*_1__| and *c*_2_ = |*C*_→^2^*m*_2__| then:

(9) $$\text{Pr}\;[𝒜(q,N)=s]=\frac{\left|N_{m_{1}}\right|+\left|N_{m_{2}}\right|}{N}+\frac{1}{2}\left(\frac{1}{c_{1}+1-\left|N_{m_{2}}\right|}+\frac{1}{c_{2}+1}\right)\left(1-\frac{\left|N_{m_{1}}\right|+\left|N_{m_{2}}\right|}{N}\right)$$

In general, we have:

**Claim 4:** Let us assume that *A* is in possession of *k* nodes *m_k_* → ^*r*_1_^ ⋯ →^*r*_*k*−2_^ *m*_2_ →^*r*_*k*−1_^ *m*_1_ and let *m_f_* and *m_b_* denote the average number of forward and backward nodes averaged over all the *k* nodes. Let *t* = *m_f_* + *m_b_* + 1. Let for 1 ≤ *i* ≤ *k*, *c_i_* = |*C*_→^2^*m*_*i*__|, then:

(10) $$\text{Pr}\;[𝒜(q,N)=s]=\frac{\mathrm{kt}}{N}+\frac{1}{k}\left(\frac{1}{c_{1}+1-(k-1)t}+\frac{1}{c_{2}+1-(k-2)t}\cdots +\frac{1}{c_{k}+1}\right)\left(1-\frac{\mathrm{kt}}{N}\right)$$

**Observations:** The probability is lowest when the adversary is actually at the base station. If the adversary has more nodes in possession, the probability increases linearly, with more success rate when the nodes are actually connected. This also shows that if a packet originates from any node that does not have a backward node, the adversary will always know the sender. This drawback can be avoided by requiring all nodes to have backward nodes. In other words, avoid a tree topology.

The above security resiliency analysis description is for route and location privacy. The security strength of identity and data privacy is mainly dependent on the encryption schemes. If encryption scheme is strong then we can achieve stronger identity and data privacy. If encryption scheme is weak then we have weak identity and data privacy.

### 5.2. Memory Consumption Analysis

Each sensor node needs to maintain one table that contains the list of neighboring nodes, their direction and their trust states as shown in Table 2. Node identity can be represent in two bytes [15, 33]. Four sets of directions can be easily represented in 2 bits. Trust calculation is based on time-based past interaction only. Therefore, the total size required to calculated trust value is 4Δ*t* bytes [22]. Here, Δ*t* represents size of time window and 4 bytes are required to store number of successful (2 bytes) and unsuccessful (2 bytes) interactions. Trust value can be represented in one byte. Therefore the size of each record is 3.25 + 4Δ*t* bytes (26 + 32Δ*t* bits). If we assume that the node has *M* neighboring nodes then the total size of the table will be *M*(26 + 32Δ*t*) bits.

In order to achieve data privacy in the presence of identity anonymity, our proposed scheme uses two keys: one Public key of the base station $k_{\mathrm{bs}}^{+}$ and other is shared secret key *k_x−bs_*. Therefore, total memory required at the sensor node for our proposed scheme is: $M\left(26+32\Delta t\right)+k_{\mathrm{bs}}^{+}+k_{x,bs}$.

Table 3 shows the memory requirement of various privacy schemes, in which *M* represents the neighborhood size, *K* represents pseudonym space, 4Δ*t* represents size of time window, and *N* is the total number of nodes in the network.

In the Phantom Flood Routing (PFR) [3] scheme, each sensor node needs to maintain the list of neighbor nodes and these neighbor nodes are divided into two sets. Here we assume that identity of a node is represented by two bytes, and set is distinguished by a single bit. So the total memory required by each node in the PFR scheme is (16+1)*M* bits. In the Phantom Single-path Routing (PSR) [4] scheme, each node maintain the list of neighbor nodes, hop count (2 bytes), and set identification (1 bit). Therefore, the total memory required by each node in the PSR scheme is (16+16+1)*M* bits. In the SAS scheme, each node needs *K*(4*M*+2*N*)+16*M* bits of memory. Here*M* represents the neighborhood size, *K* represents pseudonym space and *N* is the total number of nodes in the network. For the CAS scheme, each node requires *K*(6+7*M*)+16*M* bits of memory. (See [5] for more details about the SAS and CAS schemes.)

**Figure 5.**
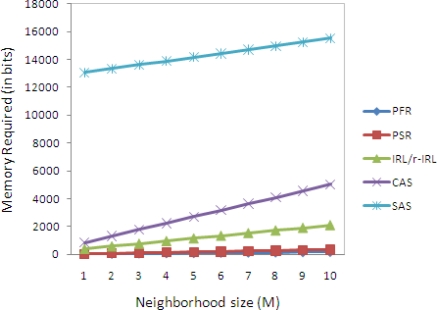
Memory consumption analysis: *N*= 100; *K*=8 bytes; Δ*t* = 5; $k_{\mathrm{bs}}^{+}=20$ bytes; *k_x−bs_* = 8 bytes.

Let us assume that the sensor node has ten neighbor nodes, then the total memory required by the sensor node by the PFR, PSR, IRL, CAS and SAS is 21.25, 41.25, 260.5, 628 and 1940 bytes respectively, as shown in Figure 5.

Additionally, cycle prevention strategy (Section 4.2) requires some short term memory to store signature of the packet for short period of time (*δt*). In our proposed schemes, signature of the packet comprises of six fields: 1) Sequence number (2 bytes), 2) previous hop identity (2 bytes), 3) next hop identity (2 bytes), 4) payload (variable size), 5) counter (2 bits), and 6) *δt* time (4 bytes). So, for each packet sensor node requires 10.25 + *Size* (payload) bytes of memory. The packet signature will be removed from the buffer after *δt* time. For example, sensor node *x* received 20 packets of equal size of payload (e.g., 10 bytes). Then, the total memory required by the sensor node is 20 × (10.25+ 10) = 405 bytes. This additional overhead does not make sensor nodes overloaded because of following reasons:

1. Generally, wireless sensor networks are event driven [34–36] or sensor nodes generate packets in periodic intervals [37, 38]. Therefore, the amount of overall traffic usually remains low.
2. In our proposed schemes, packets always follow different routes. Therefore, the probability of a single node to be overloaded is very low.

Let us assume that the amount of traffic is very high and single sensor node needs to store large amount of packets at a time. Then in order to reduce the size of memory, we can use the technique of signature generation code [39]. This technique allows us to represent single signature code in few bytes. However, this technique is based on Bloom filters [40] that require the computation of multiple hash values. This may increase the computational cost. Therefore, we need to trade off between memory and computation cost.

### 5.3. Energy Consumption Analysis

In this section, we will show the efficiency of our routing strategies with existing schemes. Energy is computed based on the communication overhead (including transmission and reception cost, path length) introduced by our proposed routing protocols and compared it with other existing schemes.

We have implemented our IRL and r-IRL routing schemes on Sensor Network Simulator and Emulator (SENSE) [41]. At the application layer we used constant bit rate component (CBR) that generate constant traffic during simulation between randomly selected source node(s) and the base station. For the simplicity, we assumed that both sensor nodes and the base station are static. Network consists of 300 sensor nodes that are organized into 15 by 20 grid manner. Other simulation parameters are given in Table 4.

We have compared our proposed IRL and r-IRL algorithms with the four variations of phantom routing schemes [3, 4] that are:

1. Phantom single path routing scheme with hop-based approach (PSR-hop).
2. Phantom single path routing scheme with sector-based approach (PSR-sec).
3. Phantom flood routing scheme with hop-based approach (PFR-hop).
4. Phantom flood routing scheme with sector-based approach (PFR-sec).

We did not compared our schemes with the SAS and CAS [5] schemes because the authors did not propose any routing strategy.

**Figure 6.**
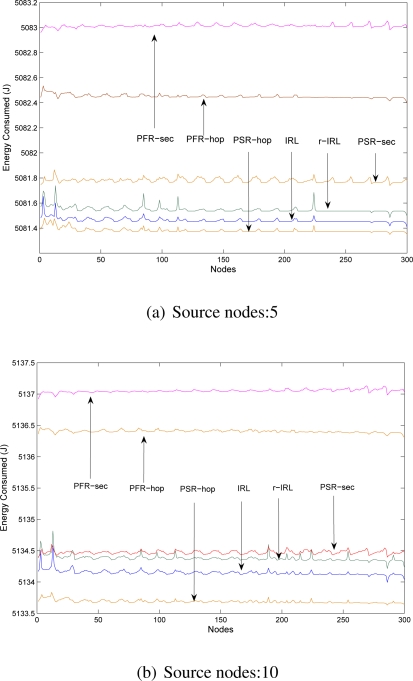
Energy consumption analysis: simulation time: 5,000.

The energy consumption analysis with different scenarios are shown in Figure 6. For the r-IRL scheme we select *r* = 3, which means a single packet will reach the destination via three different routes simultaneously. For phantom routing schemes, we select parameter *h_walk_*=10 (as recommended in [3]). Figure 6 clearly indicates that, the IRL and r-IRL schemes consume less energy as compared to the PSR-sec, PFR-hop and PFR-sec schemes but slightly consume higher energy as compared to the PSR-hop scheme. This is due to the fact that the IRL and r-IRL algorithms provides more path diversity and packets sometimes took longer paths.

### 5.4. Path Diversity Analysis

Strength of route privacy is dependent on path diversity. High path diversity provides strong route privacy and low path diversity provides weak rout privacy. Path diversity can be categorized into two types.

1. Length variation: Path could be long or short and mainly dependent on routing scheme. For example, packets always reach to the destination via shortest path. In this scheme, packets may reach to the destination via longer path if any node is not working properly within the shortest available path. With respect to the route privacy, length variation provides minimum route privacy. If we have longer paths, then it will increase time for an adversary to find out actual source node or vice versa. So, the longer path increases safety time.
2. Path variation: Each packet may follow different route. It is also dependent on routing strategy. For example, routing scheme make decision about next hop based on the energy level of neighboring nodes. With this approach, one can achieve limited path variation. With respect to the route privacy, if we have more path variation, then it will become clueless for an adversary to guess from where next packet will come.

Our proposed routing strategies (IRL and r-IRL) have both features. Because of the concept of *direction* (Section 4.1), proposed schemes provide more length variation and because of the *randomness* (Section 4.2) proposed schemes provide high path variation. Incorporation of both features offer high path diversity.

**Figure 7.**
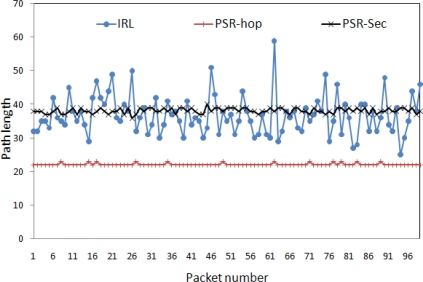
Path diversity of privacy schemes.

In order to analyze the path diversity behavior, we have organized 300 sensor nodes in a 10 by 30 grid manner. The rest of simulation parameters are given in Table 4. In the simulation, a single source node (ID: 224) generates 100 data packets for the base station. Figure 7 shows the path diversity (in terms of path length) of the IRL, PSR-hop and PSR-sec schemes. The average path taken by the PSR-hop, IRL and PSR-sec is 22.12, 36.81 and 38.17, respectively. It indicates that the IRL scheme incurs more delay as compared with the PSR-hop scheme and less delay as compared with the PSR-sec scheme. This figure also indicates that the IRL scheme has more path variation as compared with the other schemes, which creates more difficulties for the adversary to trace back the source from the captured packets.

**Figure 8.**
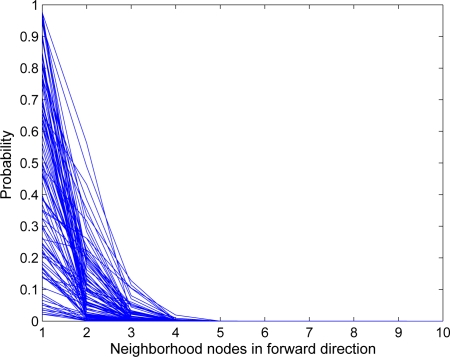
Probability of a packet to move in the backward direction.

Figure 7 also shows that some packets took longer paths in the IRL scheme as compared with others. This is due to the fact that the source or en-route node did not find any trusted node in its forward direction, so the packet is relayed back in the backward direction. If we assume that each node has *p* probability to be trusted and all probabilities are independent of each other, then the total probability *P_b_* for a node *i* to relay the packet in the backward direction is:

(11) $$P_{b}(i)=\prod_{k=1}^{m_{f}}\left(1-p_{k}\right)$$

where *m_f_* represents the number of nodes in the forward direction. Figure 8 shows the result of 100 simulation runs in which we have assumed that each node has equal probability to be trusted and un-trusted. It shows that, as the neighborhood size increases, the probability of the packet to move in the backward direction decreases sharply.

### 5.5. Discussion

From the memory, energy and path diversity analysis, we see that our solution is optimal especially with respect to the PSR-hop scheme. However, at a modest cost of memory and energy, our solutions provide full network level privacy as compared with the other existing schemes. This cost is justifiable because we have additionally achieved trustworthiness and reliability (in terms of packet reach-ability). With this level of resource consumption, our solutions can easily be used on real sensor nodes, for example, MICA2 sensor node has ATMega 128L micro controller (8 MHz @ 8 MIPS), 128 Kbyte program flash memory, 512 Kbyte measurement (serial) flash, and 4 Kbyte EEPROM [42].

## 6. Conclusions and Future work

Existing privacy schemes of WSNs only provides partial network level privacy. Providing full network level privacy is a critical and challenging issue due to the constraints imposed by the sensor nodes (e.g., energy, memory and computation power), sensor network (e.g., mobility and topology) and QoS issues (e.g., packet reach-ability and timeliness). Therefore, in this paper we proposed the first full network level privacy solution that is composed of two new identity, route and location privacy algorithms and data privacy mechanism. Our solutions provide additional trustworthiness and reliability at modest cost of energy and memory. We also proved analytically that our solutions provides protection against an adversary who is capable of performing privacy disclosure attacks such as eavesdropping and hop-by-hop trace backing.

In our future work, we will evaluate our proposed schemes from the perspective of computation cost that is required to perform encryption and random number generation.

## Figures and Tables

**Figure 3.**
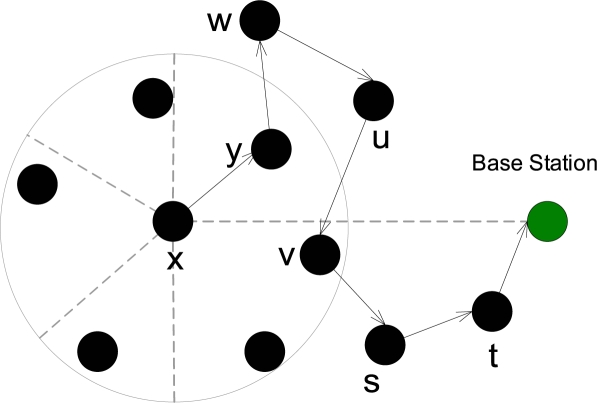
Sample routing scenario of IRL scheme.

**Table 1.** Comparison of privacy preserving schemes.

	PFR [3]	PSR [4]	SAS &CAS [5]	CEM [7]	SIGF [6]	GeRaF [8, 9]	SiFT [10]
Required information for routing	ID of destination	Routing table (e.g., destination ID, #of hops *etc*.)	Depending on a routing scheme	Depending on a routing scheme	Own, destination, & neighborhood locations	Own and destination location	Destination trajectory and own location
Transmission mechanism	1st phase: Point-to-point; 2nd phase: Broadcast	Point-to-point	Depending on a routing scheme	Depending on a routing scheme	Point-to-point	Broadcast	Broadcast
Decision place for forwarding	1st phase: Transmitter; 2nd phase: Receiver	Transmitter	Depending on a routing scheme	Depending on a routing scheme	Transmitter	Receiver	Receiver
Criteria for forwarding packet to next hop	1st phase: random; 2nd phase: flooding	1st phase: random; 2nd phase: shortest in terms of hops	Depending on a routing scheme	Depending on a routing scheme	Randomly select any trusted node lies in forwarding region	Node that is closer to the destination in terms of location	Node that is closer to the destination in terms of trajectory
Identity privacy	Not Available	Not Available	Available	Not Available	Not Available	Not Applicable	Not Applicable
Route privacy	Available	Available	Depending on a routing scheme	Depending on a routing scheme	Available	Available	Available
Location privacy	Available	Available	Not Available	Available	Available	Not Applicable	Not Applicable
Data privacy	Not Available	Not Available	Available	Available	Available	Not Applicable	Not Applicable

**Table 2.** Neighbor list table at sensor node.

Neighbor nodeID (Integer)	Direction	Past interactions based on time window						Trust value
		Successful interactions (*S_x,y_*)			Unsuccessful interactions (*U_x,y_*)			
1	*F* (00)	10	...	5	4	...	1	90
2	*B_R_* (01)	2	...	4	8	...	2	25
⋮	⋮	⋮	⋮	⋮	⋮	⋮	⋮	⋮
M	*B_L_*(11)	5	…	7	0	…	3	70

**Table 3.** Memory requirement in bits.

PFR [3]	(16+1)*M* bits
PSR [4]	(16+16+1)*M* bits
SAS [5]	*K*(4*M*+2*N*)+16*M* bits
CAS [5]	*K*(6+7*M*)+16*M* bits
IRL / r-IRL	$M\left(26+32\Delta t\right)+k_{\mathrm{bs}}^{+}+k_{x,bs}\text{bits}$

**Table 4.** Simulation parameters.

Network specific	Number of nodes	300
	Distance b/w nodes	50 units
	Mobility of nodes	zero
Node specific	Sensor node’s Initial battery	1 × 10^6^J
	Power consumption for trans.	1.6W
	Power consumption for recv.	1.2 W
	Idle power consumption	1.15W
	Carrier sense threshold	3.65*e*^−10^W
	Receive power threshold	1.55*e*^−11^W
	Frequency	9.14*e*^8^
	Trans. & Recv. antenna gain	1.0
Protocol & Application specific	Application	CBR
	Reliability param. *r* for r-IRL	3
	*h_walk_* param. for PFR & PSR	10
